# Genetic loci underlying seed size and yield-related traits in cowpea

**DOI:** 10.3389/fpls.2026.1772657

**Published:** 2026-03-16

**Authors:** Abdoul Moumouni Iro Sodo, Christian Fatokun, Bunmi Olasanmi, Patrick Obia Ongom, Ibnou Dieng, Ousmane Boukar

**Affiliations:** 1 International Institute of Tropical Agriculture (IITA), Ibadan, Nigeria; 2 Pan African University Life and Earth Sciences Institute (Including Health and Agriculture), University of Ibadan, Ibadan, Nigeria; 3 Department of Crop and Horticultural Sciences, University of Ibadan, Ibadan, Nigeria; 4 International Institute of Tropical Agriculture (IITA), Kano, Nigeria

**Keywords:** linkage mapping, quantitative trait loci, recombinant inbred lines, seed size traits, single nucleotide polymorphism

## Abstract

Understanding the genetic bases of seed size and yield-related traits in cowpea is important for enhancing the development of market demanded varieties. The objective of this study was to identify genomic regions associated with seed size and yield-related traits in cowpea. In this study, an F_6:7_ recombinant inbred line (RIL) population consisting of 248 genotypes derived from a biparental cross involving RP270 (small seed in size) and BRSImponente (extra-large seed in size), was evaluated for two consecutive years in the field and in the glasshouse to identify quantitative trait loci (QTLs) with effects on these cowpea traits. The RILs were genotyped using a 2602 cowpea mid-density SNP panel, that was filtered down to 916 informative SNPs for the linkage analysis A total of 36 QTLs were associated with seed size, and eight with grain-yield related traits. Eight of the QTLs were found to be stable for seed size because of their presence in the same regions of the genome across the four environments. Two regions on chromosomes 7 and 11 contained QTL clusters for all evaluated traits. Gene annotations, gene ontology, and available literature on the genomic regions of QTL clusters and stable QTLs revealed 13 possible genes that might be participating in the regulation of seed-related traits of cowpea. Some SNPs were linked to multiple traits thereby reinforcing evidence of pleiotropy. These SNPs serve as valuable tools for marker-assisted selection (MAS) and genomic selection (GS), offering practical applications for developing cowpea varieties with market desired seed related traits and grain yield.

## Introduction

Cowpea (*Vigna unguiculata* [L.] Walp.) is a major legume crop widely cultivated in sub-Saharan Africa (SSA), Asia, and parts of the Americas. It plays an important role in food security and nutrition, particularly in dry regions, due to its ability to thrive in harsh agroecological zones and high nutrient content (Timko and Singh, 2008; Boukar et al., 2016). Cowpea, as a legume crop, can fix up to 240 kg of nitrogen per hectare in its root nodules, providing a nitrogen benefit of 60–70 kg/ha to succeeding crops in rotation (Pule-Meulenberg et al., 2010). It is a staple crop with multiple market classes distinguished by seed coat color, seed coat texture, and size preferences, which are important determinants of consumer acceptance and market value (Ehlers and Hall, 1997; Badiane et al., 2012; Gaafar et al., 2016). While the demand for cowpea has been rising, yield enhancement activities involving variety development, seed systems, good agronomic practices, etc., are also gaining attention globally. As with many other grain crops, the acceptance of cowpea by consumers is mainly driven by the visual appearance of the grains. For the boiled grain cowpea market segment, large seed size is preferred, which leads to price premiums for large cowpea grains (Lucas et al., 2013). Across markets in various SSA communities, cowpea grain is available in a diversity of sizes, shapes, coat color patterns, and eye color. In West Africa, the two most popular grain types are medium to large white or brown with rough seed coat texture, while in the East and Southern regions of Africa, relatively smaller seeds with smooth texture and brown to red color predominate in markets (Lucas et al., 2013). Grain yield in cowpea is determined by components that include the number of peduncles per plant, the number of pods per plant, pod length, the number of branches per plant, the number of seeds per pod, and 100-seed weight (Ogunkanmi et al., 2013; Zaki and Radwan, 2022).

Seed size is an essential trait in flowering plants and plays a critical role in adaptation to the environment. Larger cowpea seeds tend to emerge earlier when sowed deep (up to 5 cm) and generate larger plants during early stages of development (Lush and Wien, 1980; Tao et al., 2017). In addition, seed size impacts yield directly while also indirectly improving stress tolerance through better competition for resources (Haig, 2013; Lo et al., 2019; Garcia-Oliveira et al., 2020). Seed length, width, thickness, and weight are parameters that determine seed size (Garcia-Oliveira et al., 2020). Studies have shown that there is a positive correlation between seed size and grain yield. Seed size and other yield components are quantitatively inherited, being controlled by polygenes and influenced by the environment. They are therefore more difficult to select for if based mainly on the phenotype (Elattar et al., 2021). Mapping genomic regions controlling seed size and yield components increases our knowledge about the genetic basis of these traits, and this will help to accelerate the efficiency of the cowpea breeding program. An important tissue that connects the pod wall and seed and provides a pathway for delivering nutrients and photosynthates to the developing embryo is the seed hilum (Hardham, 1976; Thorne, 1981). The major and minor hilum axis lengths have positive correlations with protein content and individual seed weight in soybean (Barion et al., 2016). Furthermore, seeds with intact hilum exhibit relatively high seed vigor, while those with injured hilum show reduced nutrient flow to the seed and produce poor-quality seeds that are more likely, when sown, to experience poor germination and yield losses. Infection of the seeds with damaged hilum by pathogens is a possibility (Hsieh et al., 2005; Kumar et al., 2019; Brijesh et al., 2022). Importantly, the hilum facilitates water uptake during seed germination (Pietrzak et al., 2002; Zhang et al., 2004; Jaganathan et al., 2019). Hilum attributes may have been selected during the domestication process, as this tissue functions as a hygroscopically activated valve in the impermeable epidermis, which plays a crucial role in seed dormancy (Hyde, 1954; Lush and Evans, 1980). Overall, previous studies have shown that seed-related traits, including seed hilum size, significantly and positively impact seed weight and seed quality (Zhao et al., 2021). However, despite the hilum playing a significant role in the development of vigorous seedlings, it seems plant breeders have not paid much attention to it. There is a paucity of evidence indicating the genetic factors underlying the role of the hilum in improving cowpea grain yield and seed quality under field conditions. Available literature reveals that a relatively small number of QTL analyses have been carried out for the purpose of genetic dissection of grain yield and seed size-related traits in cowpea (Andargie et al., 2014; Lo et al., 2018; Garcia-Oliveira et al., 2020; Sodo et al., 2025). Reports have not been consistent or similar across studies due to differences in parental lines, their genetic backgrounds, marker densities, population sizes, and phenotyping conditions. Although some regions have been identified with identical markers associated with seed size QTLs, many loci remain unique to specific populations, highlighting the need for further studies across multiple environments and populations to validate these QTLs. The objective of the present study was to identify QTLs controlling cowpea seed size and yield related traits by genotyping, using mid-density SNP markers, a RIL population derived from the bi-parental cross RP270 x BRSImponente grown in the field and glasshouse.

## Materials and methods

### Genetic materials

A recombinant inbred line (RIL) population comprising 248 entries was developed from the cross between RP270 and BRSImponente using the single-seed descent breeding method. The RP270 is a small, white-seeded landrace line (approx. 15.5 g/100 seed weight) collected from the Republic of Togo for a genetic diversity study, with smooth coat texture, no eye color an indeterminate prostrate growth habit. The BRSImponente is a large, white-seeded (approx. 29.5 g/100 seed weight), improved breeding line from Brazil, having brown eyes, a rough seed coat texture, and a semi-erect growth habit.

### Phenotyping

The RILs, along with their two parents, were evaluated in the experimental field of IITA, Ibadan (7.50250° N, 3.89411° E), Nigeria, for two cropping seasons. The seeds were sown on 13^th^ October 2022 and 4^th^ September 2023 following ploughing and harrowing on 28^th^ August 2023 and 3^rd^ September 2024. The experimental design was an incomplete lattice composed of fourteen blocks, with 18 RILs per block. Each experimental plot consisted of three rows of 2.0 m in length, with a spacing of 1.0 m between rows and 0.25 m within rows. The field trials were managed according to the recommended agronomic practices to ensure optimum plant growth and development. In the field, the eight plants in the middle rows were selected in each plot, and the following traits were measured: number of branches per plant (NBrch), number of peduncles per plant (Nped), peduncle length (PedLt), pod length (PodLt), number of pods per plant (NPod), and number of seeds per pod (NSP). Peduncle and pod lengths were recorded using a flexible measuring tape. Peduncle length was recorded by measuring the distance from the point of peduncle attachment to the stem node to where the first flower bud appeared. The number of seeds per pod was recorded on ten randomly selected mature pods. In the glasshouse, plantings were carried out on 30^th^ May 2022 and 11^th^ November 2024 using plastic pots, each filled with five kg of sterilized topsoil. Three seeds of each RIL were sown per pot, and seedlings were thinned to one plant per pot two weeks after sowing. Pots were watered each day. After harvesting, the pods were threshed, and seeds dried to constant moisture level in the glasshouse. Physically damaged, diseased, or infested seeds were eliminated prior to weighing 100-seed weight (HSW). Ten seeds were selected for measurements of the seed size-related traits, including seed length (SL) which is the longest distance parallel to the seed hilum; seed width (SW) which is the distance across the seed; seed thickness (ST) measured as the distance between the hilum and the opposite side; seed hilum length (SHL) i.e. the distance from one end of the hilum to the other; and seed hilum width (SHW) measured as the distance across the hilum at its widest point. Measurements were carried out using a Vernier caliper according to Kaushik et al. (2007). The seed hilum area (SHA) was determined by considering the hilum as an ellipse and using the hilum’s length and width to calculate its area using the formula for area of an ellipse: SHA = SHL × SHW × π/4 (Zhao et al., 2021).

### Phenotypic data analysis

A mixed-effects linear model (MLM) was employed to analyze the data. Analysis of variance (ANOVA) was conducted to assess the significance of fixed effects, while Best Linear Unbiased Predictors (BLUPs) were extracted to estimate random effects and genotypes’ genetic values using the lme4 package implemented in R (Ohmomo et al., 2022). Descriptive statistics such as mean, range, coefficient of variation (CV %), skewness, and kurtosis for the measured seed traits in the RIL population, including the parental genotypes, were estimated. Broad-sense heritability (H²) was calculated using the following equation:


$$H^{2}=\sigma_{G}^{2}/\;(\sigma_{G}^{2}+\sigma_{GE}^{2}/n\;+\sigma_{e}^{2}/nr)$$

where σ^2G^ represents the genotypic variance, σ^2GE^, the variance of the genotype-by-environment interaction, σ^2e^, the error variance estimated from the ANOVA, n is the number of environments, and r is the number of replications within the environment. The Pearson correlation coefficient (r) between two seed and yield-related traits was computed. The combined BLUEs across two years for yield related traits were used for QTL analysis, while for seed size–related traits, the individual BLUEs from each year (field and glasshouse) were used for QTL analysis.

### Genotyping linkage map construction

Two weeks after seedling emergence, newly expanded young middle leaflets of the trifoliate leaf were sampled per plant and placed in ziplock bags containing silica gel for desiccation according to Intertek Agritech laboratory’s protocol (Intertek-Agritech, 2016). Genotyping was conducted using the cowpea mid-density DArTag SNP panel that contains 2,602 quality SNPs selected based on the following criteria: (i) missing data rate< 5%, (ii) minor allele frequency (MAF) > 0.20, and (iii) uniform distribution across the genetic linkage groups to ensure adequate genome coverage (Ongom et al., 2024). This mid-density panel was used to genotype 250 DNA samples (248 RILs and 2 parents) reported in the present study. DArTag genotyping was performed using targeted molecular probes designed to capture short genomic regions containing sequence polymorphisms. These regions were amplified, and sample-specific barcodes were incorporated during library preparation. The libraries were sequenced on Illumina HiSeq 2500/Novaseq platforms, generating approximately 1.2 million reads per sample. Sequence data were processed using DArT PL’s proprietary pipeline, which involved alignment to the cowpea reference genome of the IITA cowpea variety IT97K-499–35 reference genome, which can be accessed on Phytozome [https://phytozome-next.jgi.doe.gov/info/Vunguiculata_v1_1 *Vigna unguiculata* v1.1 (Lonardi et al., 2019)]. Allele calling was conducted based on the relative counts of alternative alleles for each marker in each sample. The 2,602 SNPs were filtered further before conducting linkage mapping leaving 916 SNPs. The filtering criteria included: 20% missing genotype, segregation distortion, and marker redundancy. Data filtering protocols and linkage map construction were consistent with those applied in our earlier study (Sodo et al., 2025); detailed methodologies are described therein.

### QTL analysis and putative gene prediction

A constructed linkage map was used to identify quantitative trait loci (QTLs) for all assessed traits via inclusive composite interval mapping (ICIM-ADD) method using IciMapping v.4.2 software (Intertek-Agritech, 2016). During the QTL mapping process, several critical parameters were set. These included 1000 shuffles for estimating the critical values of LOD at p = 0.05 (P-value) for the permutation option to declare the presence of a significant main-effect QTL. The QTLs explaining 10% or more of the phenotypic variation (PVE≥ 10%) were considered major, whereas those explaining less than 10% were classified as minor. The RILs were evaluated for seed size for two years in the field and in the glasshouse, which were considered four different environments. For yield-related traits, we used combined BLUEs across two years because these traits are highly controlled by environmental factors and genotype × environment (GE) interactions, and combining the data increases the accuracy of QTL identification. While seed size traits are generally more stable across environments; therefore, we conducted QTL analysis using individual BLUEs from each year (field and glasshouse) to capture potential environment-specific QTLs and better understand the expression of these traits under different conditions. According to Luo et al. (2023), QTLs should be identified across a minimum of two environments to be considered stable. The QTLs were arranged by chromosome and marker interval positions. With gene mining and annotation, the genes were identified within the interval of the flanking markers for stable QTLs from the cowpea reference genome (http://phytozome.jgi.doe.gov/). The putative genes were identified using the relevant literature and related gene functions.

### Haplotype estimation and SNP effect prediction

The haplotype associated with a significant QTL was constructed using the “ggsignif” and “ggpubr” packages in R (Yin, 2019).The haplotype sequences were determined based on the RILs utilized for testing and identification. Variant effect predictions were estimated using the adjusted posterior probability to identify markers with high segregation. Marker effects were then visualized using the ggplot2 package in R.

## Results

### Phenotypic analysis

**Figure 1:**
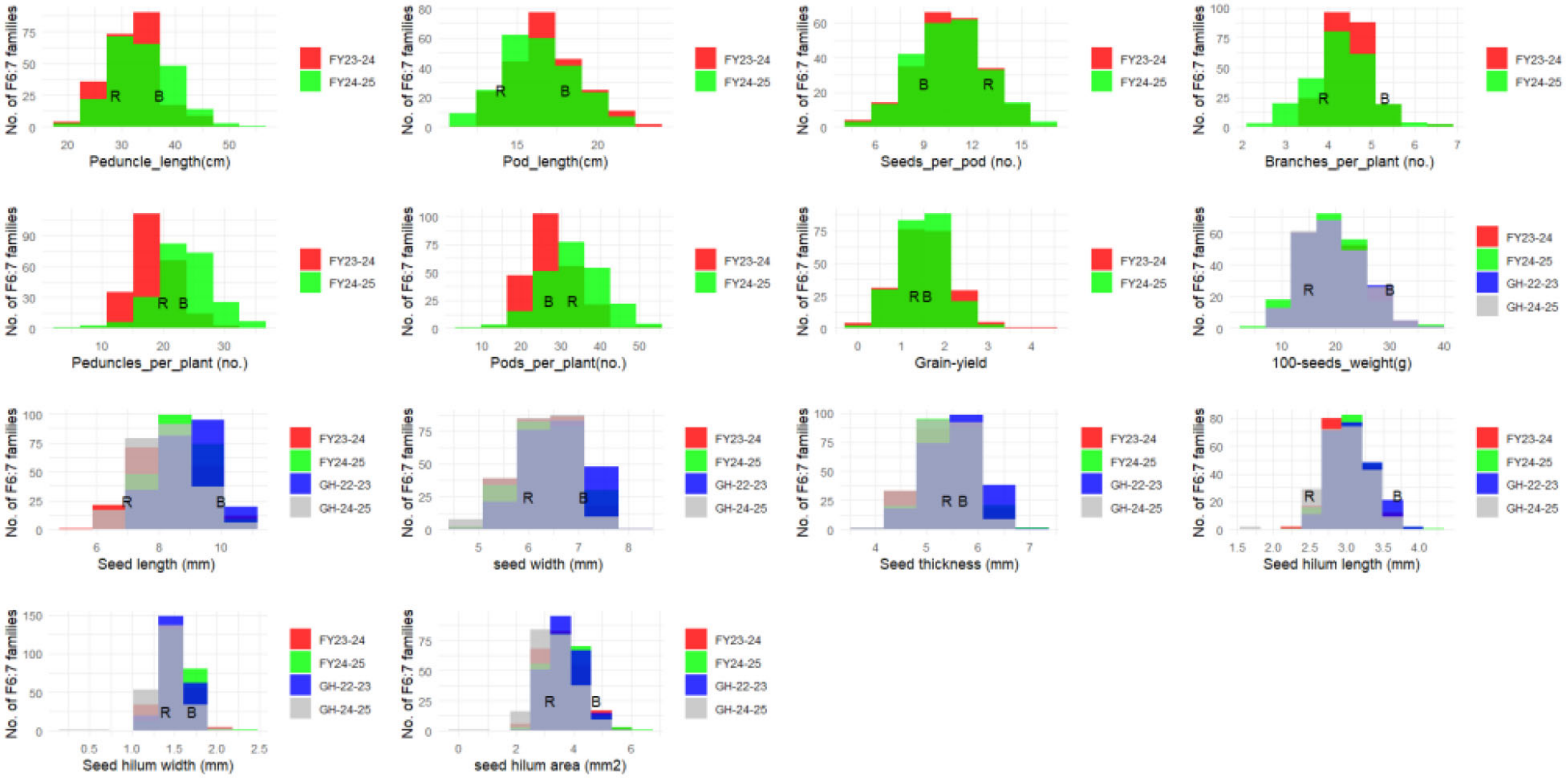
Distribution of seed size and yield-related traits of RP270× BRSImponente cowpea RIL population grown under field and glasshouse conditions (FY-23-24, FY24-25, GH-22-23, and GH-24-25). Parental means are indicated with R for RP270 and B for BRSImponente. PedLt, peduncle length; PodLt, pod length; NSP, number of seeds/pods; NBrch, Number of branches/plants; Nped, number of peduncles/plant; Npod, number of pods/plant; HSW, hundred seed weight; GY, Grain yield/plant.

**Figure 2:**
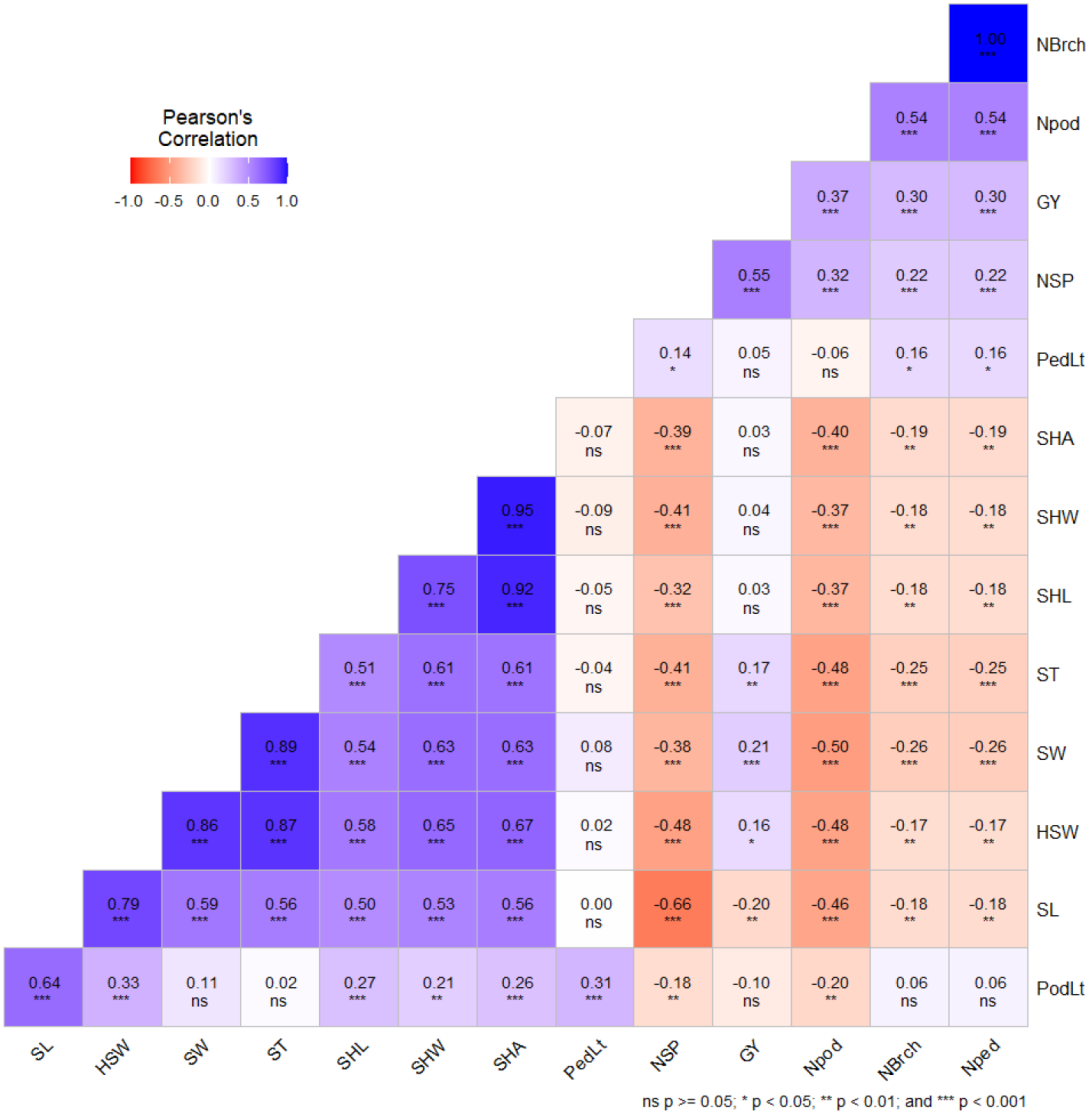
Correlation coefficients among seed size related traits and grain yield. PedLt, Peduncle length; PodLt, pod length; NSP, number of seeds/pod; NBrch, Number of branches/plant; Nped, number of peduncles/plant; Npod, number of pods/plant; HSW, hundred seed weight; GY, Grain yield/plant; CV, coefficient of variance; SL, seed length; SW, Seed width; ST, seed thickness; SHL (seed hilum length, mm), SHW (seed hilum width, mm), and SHA (seed hilum area, mm^2^). *, **and *** refers to statistical significance at the probability levels of 0.05, 0.01 and 0.001 respectively.

The mean, minimum, and maximum values; standard error; skewness; kurtosis; heritability (H²); and coefficients of variation (CV %) associated with each of the measured traits are presented in **Table 1**. The results showed that the two parents contrasted in the traits measured (**Figure 1**; **Table 1**). Significant variations were observed between parents and among the RILs for the evaluated traits. According to the normality test, absolute values of skewness and kurtosis for all the traits during the different years were all less than one. The results indicated that the segregation patterns of all the measured traits fit a normal or skew-normal distribution, exhibiting typical quantitative genetic pattern influenced by multiple genes. Some of the RILs exhibited lower or higher values than the parental lines, implying that transgressive segregation was widely present in this mapping population (**Figure 1**; **Table 1**). Frequency distributions showed no significant deviations from expectation for all traits across the four different environments (**Figure 1**). These, therefore, showed that this RIL population is suitable for QTL analysis. Broad-sense heritability (H^2^) ranged from 0.40 to 0.97, indicating high heritability and suggesting that genetic variance was superior to other variances, and this mapping population was appropriate for high-efficiency selection for the measured traits in cowpea. Significant and positive correlations were found between hundred seed weight and the other measured seed-related traits (SL, SW, ST, SHL, SHW, and SHA) with correlation coefficients (r²) ranging from 0.58 to 0.87 (**Figure 2**). Hundred seed weight displayed a significant and negative correlation with the number of seeds per pod with correlation coefficient of r = -0.48. Mean grain yield was correlated with the number of seeds per pod, hundred seed weight, number of peduncles per plant, number of pods per plant, number of branches per plant other seed size traits (**Figure 2**).

**Table 1:** Phenotypic variation and genetic analysis of the evaluated traits in F6:7 recombinant inbred lines (RILs) and their parents (RP270, BRSIM) evaluated under field and glasshouse conditions.

Traits	Seasons	Parents (mean)		248 F6-RILS					
		RP270	BRSIM	Range	Mean	SE	Skew	CV %	H_2_ %
PedLt	FY-23-24	29.0	37.4	15.2-54.1	32.2	0.21	0.2	17.9	0.80
	FY-24-25	25.5	34.6	19.1-53.74	33.8	0.37	0.3	18.2	0.78
PodLt	FY-23-24	14.7	18.4	11.1 -26.3	16.9	0.09	0.5	14.1	0.94
	FY-24-25	13.4	18.6	11.84-22.31	16.4	0.14	0.3	15.2	0.87
NSP	FY-23-24	13.2	8.8	3.4-17.2	10.6	0.09	-0.1	23.0	0.91
	FY-24-25	12.9	10.5	4.8-14.67	10.4	0.12	-0.1	21.4	0.79
NBrch	FY-23-24	3.6	5.7	2.5-7-3	4.5	0.03	0.1	15.4	0.67
	FY-24-25	4.6	4.8	3.5-6.0	4.6	0.03	0.1	13.6	0.40
Nped	FY-23-24	20.4	20.0	6-40.6	18.6	0.18	0.6	24.5	0.71
	FY-24-25	22.3	26.1	5.2-35.5	23.3	0.31	-0.3	26.1	0.68
Npod	FY-23-24	32.2	29.6	9.9-63.9	28.1	0.28	0.6	26.9	0.77
	FY-24-25	33.9	36.1	6.2-52.0	33.1	0.49	-0.2	27.8	0.77
GY	FY-23-24	1.6	1.7	0.08-2.9	1.3	0.02	0.1	37.4	0.87
	FY-24-25	1.5	1.8	0.1-3.2	1.5	0.03	0.0	41.3	0.82
100SW	FY-23-24	14.6	30.0	5.84-39.6	18.4	0.22	0.6	30.9	0.97
	FY-24-25	15.4	30.2	5.8-37.98	19.0	0.35	0.4	29.8	0.96
	GH-22-23	14.63	30.0	5.6-39.9	18.87	0.2	0.6	31.9	_
	GH-24-25	15.4	29.9	7.5-38.7	19.50	0.2	0.6	33.4	_
SL	FY-23-24	7.7	10.6	6.2-11.1	8.38	0.04	0.23	12.0	0.96
	FY-24-25	7.49	10.35	6.7-11.1	8.44	0.05	0.39	10.40	0.94
	GH-22-23	7.65	10.6	6.16-11.07	8.90	0.04	0.30	12.45	_
	GH-24-25	7.3	11.0	6.0-10.5	8.30	0.06	0.15	13.4	_
SW	FY-23-24	6.1	7.2	4.7-7.8	6.36	0.02	-0.07	9.6	0.96
	FY-24-25	6.23	7.18	4.89-8.0	6.44	0.04	-0.06	9.62	0.96
	GH-22-23	6.06	7.21	4.72-7.97	6.60	0.02	-0.06	9.78	_
	GH-24-25	6.5	7.4	4.8-7.9	6.30	0.04	-0.20	9.2	_
ST	FY-23-24	5.4	5.7	3.7-7.1	5.39	0.02	0.03	9.7	0.95
	FY-24-25	5.5	5.8	3.76-7.13	5.59	0.02	0.05	9.83	0.96
	GH-22-23	5.5	5.77	4.09-7.14	5.44	0.03	0.14	9.38	_
	GH-24-25	5.5	5.6	4.0-6.7	5.31	0.03	-0.11	10.2	_
SHL	FY-23-24	2.8	3.6	2.2-4.4	3.02	0.01	0.34	10.4	0.93
	FY-24-25	2.7	3.78	2.16-4.5	3.23	0.01	0.36	11.41	0.94
	GH-22-23	2.8	3.6	2.2-4.4	3.02	0.01	0.34	10.4	_
	GH-24-25	2.4	3.8	1.6-3.7	3.00	0.02	-0.52	11.7	_
SHW	FY-23-24	1.5	1.7	0.9-2.2	1.50	0.01	0.54	12.9	0.93
	FY-24-25	1.50	1.75	1.16-2.24	1.55	0.01	0.58	12.88	0.91
	GH-22-23	1.45	1.68	0.95-2.22	1.50	0.01	0.54	13.73	_
	GH-24-25	1.5	1.8	0.20-2.04	1.43	0.01	-0.77	11.0	_
SHA	FY-23-24	3.2	4.7	1.6-6.9	3.58	0.03	0.67	21.2	0.94
	FY-24-25	3.25	4.73	1.66-6.88	3.61	0.03	0.70	22.21	0.95
	GH-22-23	3.28	4.93	2.22-6.66	3.72	0.04	0.83	20.09	_
	GH-24-25	3.1	4.3	0.25-5.6	3.40	0.05	-0.08	18.6	_

FY, Field year; GH, Glass house; PedLt, Peduncle length; PodLt, pod length; NSP, number of seeds/pod; NBrch, Number of branches/plant; Nped, number of peduncles/plant; Npod, number of pods/plant; HSW, hundred seed weight; GY, Grain yield/plant; CV, coefficient of variance; SL, seed length; SW, Seed width; ST, seed thickness; SHL (seed hilum length, mm), SHW (seed hilum width, mm), and SHA (seed hilum area, mm^2^), coefficient of variance (CV%), broad-sense heritability (H^2^%).

### Genetic linkage map

Following quality control of genotypic data, 916 high-quality SNP markers with confirmed positions were used to construct a linkage map covering 711.70 cM of the cowpea genome (**Supplementary Table 1**), with a cumulative average distance of 0.80 cM between adjacent markers. The number of SNP markers mapped on each of the eleven *V. unguiculata* linkage groups ranged from 38 for Vu08 to 118 for Vu03, spanning from 52.55 cM for Vu02 to 89.57 cM for Vu05, with a cumulative average of 64.70 cM.

### Identification of QTLs

At the first stage, a QTL analysis was performed to identify single-locus QTLs affecting the evaluated traits. Using interval composite mapping under the additive model (ICIM-ADD) and LOD thresholds calculated based on 1000 permutations, a total of 44 main effect QTLs associated with PedLt, PodLt, NSP, NBrch, Nped, Npod, HSW, GY, SL, SW, ST, SHL, SHW, and SHA were detected on six chromosomes (Vu03, Vu04, Vu07, Vu08, Vu09, and Vu11) (**Table 2**; **Supplementary Figure 1**). Out of the 44 QTLs identified here, 18 were novel (**Table 3**). Phenotypic variance explained by the identified QTLs ranged from 4.2% to 14.7%, with LOD values of between 3.0 and 7.9. One QTL was detected for PedLt on Vu09 which explained 7.7% of the phenotypic variance. Additive effects value indicated that the PedLt-increasing QTL was contributed by BRSImponente alleles. Regarding PodLt, two QTLs, *qPodLt-4–1* and *qPodLt-8-1*, were identified on Vu04 and Vu08, respectively. These QTLs explained a phenotypic variance of 9.20% and 4.8% with LOD scores of 5.7 and 3.0, respectively. The observed positive and negative additive values indicated that both parents contributed alleles, which increased PodLt in the RILs. One QTL for NSP was identified on Vu03, explaining 7.5% of phenotypic variation with a positive additive value, which indicates that alleles from parent RP270 contributed to the increase of this trait. One QTL for NBrch was detected on Vu11, explaining 8.5% of the phenotypic variance. Additive effect values indicated that the QTLs contributing to the increase were contributed by RP270 alleles. A significant QTL was identified on Vu07for Nped with a LOD score of 4.4 and explaining 8.5% of phenotypic variation. Remarkably, a co-localized QTL (*qNPod-7-1*) was identified for Npod at an identical region of Vu07 as Nped. This colocalization suggests potential pleiotropic effects or tight linkage between genes controlling the number of peduncles and pods per plant in cowpea, providing valuable targets for marker-assisted selection in breeding programs. Regarding HSW, a stable QTL, *qHSW-7-1*, across four distinct environments was located on Vu07, flanked by markers 2_19423 and 2_18315, with LOD scores ranging from 3.1 to 4.29, explaining between 6.5% and 8.81% of phenotypic variation. As for SL, six single-locus QTLs were found on Vu03, Vu04, Vu08, and Vu11. These QTLs explained phenotypic variances ranging from 4.2% to 8.5%, with LOD scores between 3.0 and 6.3 in the four different environments. The QTL, *QSL-8–1* for SL, was found in Chr8 at two environments. For SW, one major QTL, *qSW-7-1*, was identified on Vu07 across three of the four environments used, explaining phenotypic variation ranging from 13.3% to 14.74% with LOD scores between 7.7 and 7.9. One QTL for ST was found in the same chromosome region across all environments and explained phenotypic variance between 6.2% and 8.6%. For seed hilum length, width, and area, five, five, and eight main-effect QTLs were detected, respectively, on Vu07and Vu11, with two, three, and three identified as stable QTLs. The LOD values for these stable QTLs ranged from 3.1 to 7.1.

**Table 2:** List of putative QTLs for seed- and yield related traits.

Traits	QTLs	Chr	Pos	L-marker	R-marker	LOD	PVE (%)	Add	Env.
PedLt	*qPedLt-9-1*	Vu09	82	2_24747	2_22572	3.9	7.7	1.6	Combined seasons
PodLt	*qPodLt-4-1*	Vu04	17	2_13744	2_18697	5.7	9.2	-0.7	Combined seasons
PodLt	*qPodLt-8-1*	Vu08	69	2_21200	2_12563	3.0	4.8	0.5	Combined seasons
NSP	*qNSP-3-1*	Vu03	4	2_48250	2_28395	3.9	7.5	0.6	Combined seasons
NBrch	*qNBrch-11-1*	Vu11	41	2_11281	2_24110	4.3	8.5	-0.2	Combined seasons
Nped	*qNPed-7-1*	Vu07	37	2_42453	2_09465	4.4	8.0	1.0	Combined seasons
Npod	*qNpod-7-1*	Vu07	37	2_42453	2_09465	4.8	7.9	1.8	Combined seasons
GY	*qGY-7-1*	Vu07	51	2_03283	2_54231	5.0	9.4	-0.2	Combined
	*qHSW-7-1*	Vu07	44	2_19423	2_18315	3.5	6.5	-1.4	FY-23-24
HSW	*qHSW-7-1*	Vu07	44	2_19423	2_18315	4.3	8.8	-1.6	FY-24-25
	*qHSW-7-1*	Vu07	44	2_19423	2_18315	3.1	6.7	-1.4	GH-22-23
	*qHSW-7-1*	Vu07	44	2_19423	2_18315	3.2	6.7	-1.4	GH-24-25
	*qSL-11-1*	Vu011	0	2_25124	2_00076	3.0	6.2	0.3	FY-23-24
SL	*qSL-4-1*	Vu04	17	2_13744	2_18697	4.1	6.2	-0.2	FY-24-25
	*qSL-8-1*	Vu08	71	2_34466	2_20561	5.0	7.7	0.3	FY-24-25
	*qSL-3-1*	Vu03	81	2_10058	2_32723	3.2	4.2	-0.2	GH-22-23
	*qSL-4-1*	Vu04	18	2_40965	2_20961	5.7	7.6	-0.3	GH-22-23
	*qSL-8-1*	Vu08	71	2_34466	2_20561	6.3	8.5	0.3	GH-22-23
	*qSW-7-1*	Vu07	57	2_32831	2_29015	7.9	13.4	-0.2	FY-23-24
SW	*qSW-7-1*	Vu07	57	2_32831	2_29015	7.7	14.7	-0.2	FY-24-25
	*qSW-7-1*	Vu07	57	2_32831	2_29015	7.8	13.3	-0.2	GH-22-23
	*qSW-7-1*	Vu07	44	2_19423	2_18315	6.1	11.6	-0.2	GH-24-25
	*qST-7-1*	Vu07	44	2_19423	2_18315	3.7	8.6	-0.1	FY-24-25
ST	*qST-7-1*	Vu07	44	2_19423	2_18315	3.4	6.8	-0.1	FY-23-24
	*qST-7-1*	Vu07	44	2_19423	2_18315	3.8	7.5	-0.1	GH-22-23
	*qST-7-1*	Vu07	44	2_19423	2_18315	3.2	6.2	-0.1	GH-24-25
	*qSHL-11-1*	Vu011	10	2_08890	2_54826	5.8	11.8	0.1	FY-23-24
	*qSHL-11-1*	Vu011	10	2_08890	2_54826	6.3	12.2	0.1	FY-24-25
	*qSHL-11-1*	Vu011	7	2_04315	2_29211	7.1	12.7	0.1	GH-22-23
SHL	*qSHL-7-1*	Vu07	49	2_34565	2_03283	3.4	6.6	-0.1	GH-24-25
	*qSHL-11-1*	Vu011	7	2_04315	2_29211	5.6	11.2	0.1	GH-24-25
	*qSHW-7-1*	Vu07	56	2_20060	2_55172	3.1	7.0	0.0	FY-23-24
	*qSHW-11-1*	Vu011	10	2_08890	2_54826	3.3	7.4	0.0	FY-23-24
	*qSHW-7-1*	Vu07	56	2_20060	2_55172	3.2	7.6	0.0	GH-24-25
SHW	*qSHW-11-1*	Vu011	10	2_08890	2_54826	3.3	7.6	0.0	GH-24-25
	*qSHW-7-1*	Vu07	56	2_20060	2_55172	4.5	10.3	0.0	GH-22-23
	*qSHA-7-1*	Vu07	44	2_19423	2_18315	3.1	7.1	-0.2	FY-23-24
	*qSHA-11-1*	Vu011	10	2_08890	2_54826	4.2	9.3	0.2	FY-23-24
SHA	*qSHA-7-1*	Vu07	58	2_40009	2_51871	3.2	6.4	-0.2	GH-24-25
	*qSHA-11-1*	Vu011	10	2_08890	2_54826	4.9	9.6	0.2	GH-24-25
	*qSHA-7-1*	Vu07	44	2_19423	2_18315	3.6	8.0	-0.2	GH-22-23
	*qSHA-11-1*	Vu011	10	2_08890	2_54826	3.8	8.2	0.2	GH-22-23
	*qSHA-7-1*	Vu07	49	2_34565	2_03283	3.3	7.2	-0.2	GH-24-25
	*qSHA-11-1*	Vu011	8	2_27634	2_26151	4.2	9.5	0.2	GH-24-25

FY, Field year; GH, Glass house; PedLt, Peduncle length; PodLt, pod length; NSP, number of seeds/pod; NBrch, Number of branches/plant; Nped, number of peduncles/plant; Npod, number of pods/plant; HSW, hundred seed weight; GY, Grain yield/plant; CV, coefficient of variance; SL, seed length; SW, Seed width; ST, seed thickness; SHL (seed hilum length, mm), SHW (seed hilum width, mm), and SHA (seed hilum area, mm^2^).

**Table 3:** Summary of QTL comparison.

Traits	QTL in Present study		Pos (bp)	QTL in Previous studies			Ref
	QTLs	Chr		QTL	Chr	Pos (bp)	
PedLt	*qPedLt-9-1*	Vu09	955391	*qPedLt-9-1*	Vu09	1587282	(Sodo et al., 2025)
PodLt	*qPodLt-4-1*	Vu04	38745770	*qPodLt-4-1*	Vu04	5307765	(Sodo et al., 2025)
PodLt	*qPodLt-8-1*	Vu08	37396761	qPodLt-8-1	Vu08	37286724	(Sodo et al., 2025)
NSP	*qNSP-3-1*	Vu03	63861445		Vu03		(Sodo et al., 2025)
NBrch	*qNBrch-11-1*	Vu11	3871195	*qNBrch-11-1*	Vu11	33701608	(Sodo et al., 2025)
Nped	*qNPed-7-1*	Vu07	28483321	*qNped-7-1*	Vu07	21498532	(Sodo et al., 2025)
Npod	*qNpod-7-1*	Vu07	28483321	qNpod-7-1	Vu07	21498532	(Sodo et al., 2025)
GY	*qGY-7-1*	Vu07	23239607		Vu07		(Sodo et al., 2025)
HSW	*qHSW-7-1*	Vu07	25863610	*qHSW-7-1*	Vu07	21498532	(Sodo et al., 2025)
	*qSL-11-1*	Vu011	41324185		Vu011		Novel
SL	*qSL-4-1*	Vu04	38745770		Vu04		Novel
	*qSL-8-1*	Vu08	37708466	*qSL-8-1*	Vu08	14084982	(Garcia-Oliveira et al., 2020)
	*qSL-3-1*	Vu03	48394714	*qSL-3-1*	Vu03	25364800	(Garcia-Oliveira et al., 2020)
	*qSL-4-1*	Vu04	37980390		Vu04	–	Novel
SW	*qSW-7-1*	Vu07	19905277		Vu07		Novel
	*qSW-7-1*	Vu07	25863610	–	Vu07		Novel
ST	*qST-7-1*	Vu07	25863610		Vu07		Novel
SHL	*qSHL-11-1*	Vu011	38187692		Vu011		Novel
	*qSHL-11-1*	Vu011	39649093		Vu011		Novel
	*qSHL-7-1*	Vu07	23705735		Vu07		Novel
	*qSHL-11-1*	Vu011	39649093	–	Vu011		Novel
SHW	*qSHW-7-1*	Vu07	21498532		Vu07		Novel
	*qSHW-11-1*	Vu011	38187692	–	Vu011		Novel
	*qSHA-7-1*	Vu07	25863610		Vu07		Novel
SHA	*qSHA-7-1*	Vu07	14097900		Vu07		Novel
	*qSHA-7-1*	Vu07	23705735		Vu07		Novel
	*qSHA-11-1*	Vu011	38804016	–	Vu011		Novel

**Figure 3:**
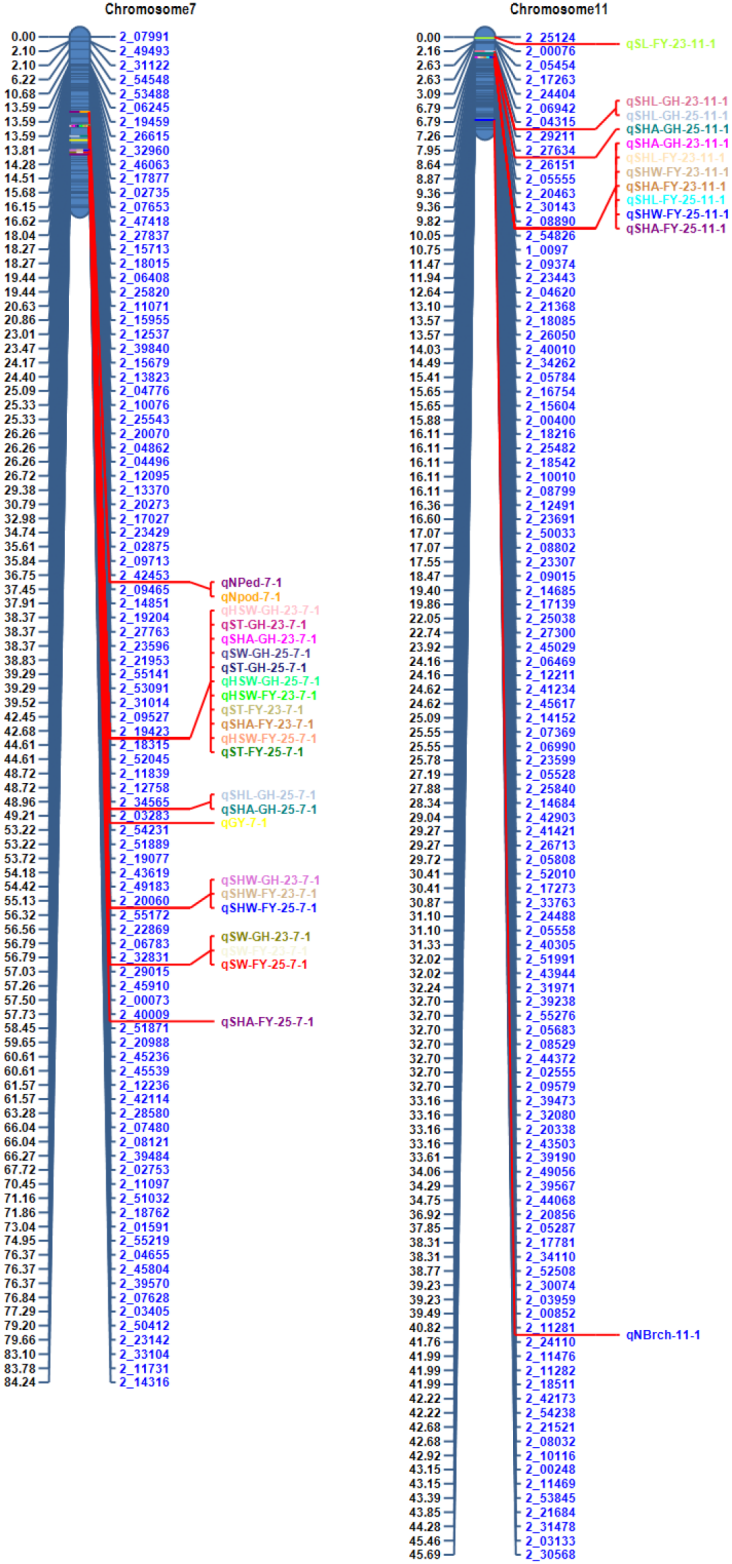
QTL hotspots/cluster mapped on two chromosomal regions.

**Figure 4:**
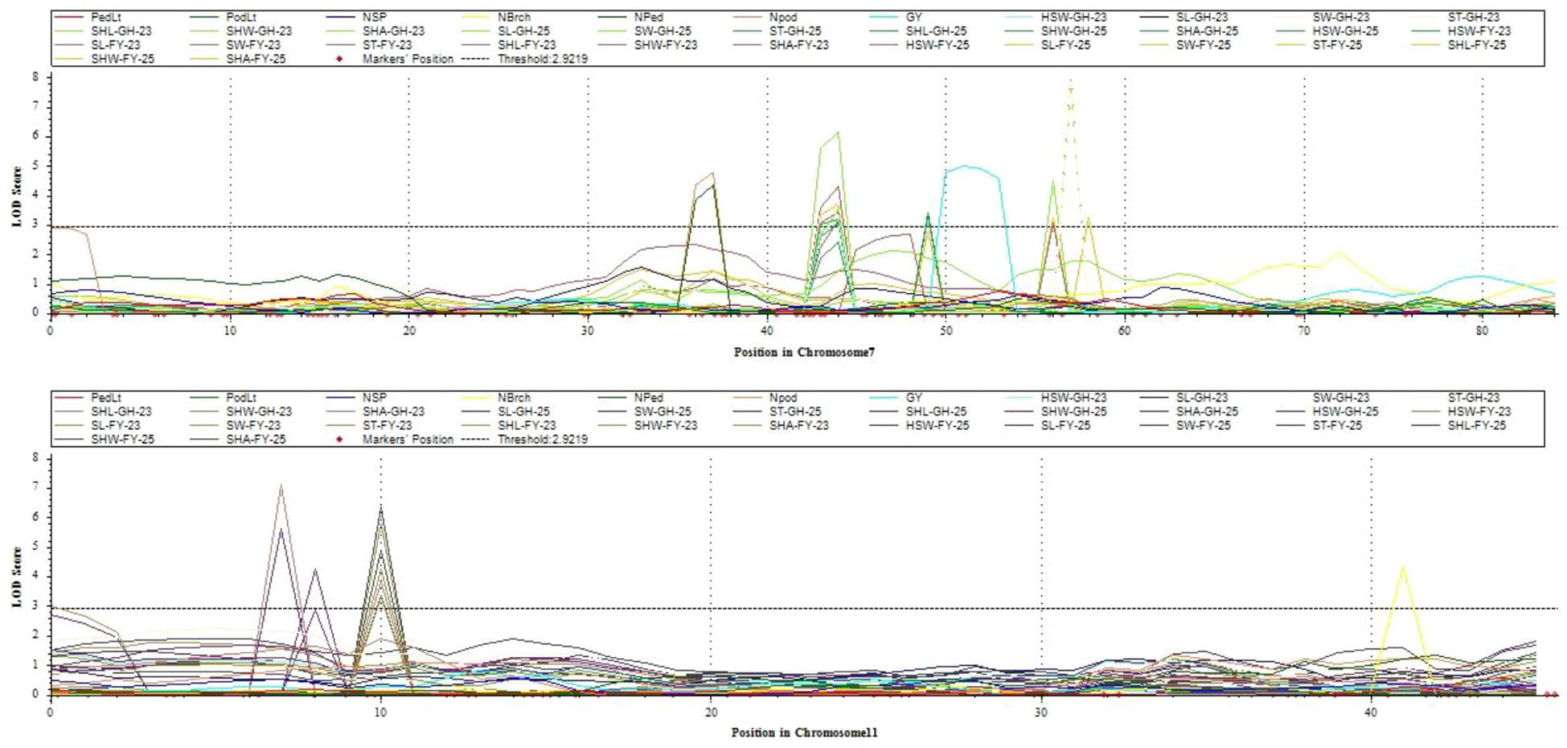
Manhattan plots of QTL clusters for measured traits. Each plot displays the LOD scores of genetic markers along Vu07 and Vu011 for multiple traits in different environments. Peaks represent potential QTLs, with significant QTLs identified by those exceeding the LOD score threshold (black dashed line).

Two significant QTL hotspots were identified on Vu07 and Vu11 (**Figure 3**). These genomic regions contain clusters of co-localized QTLs that were each consistently observed in more than one environment. These QTL clusters are likely to represent regions harboring major effect genes that control multiple traits. The co-location of the QTLs suggests either pleiotropic effects of single genes or tightly linked genes affecting seed related traits. The Manhattan plots (**Figure 4**) highlight significant QTL clusters identified on Vu07 and Vu11, detected for a multiple seed-related and agronomic traits evaluated in four environments. These QTL clusters, along with other stable QTLs identified in our study, served as reference regions for identifying potential candidate genes controlling seed size traits and were used as target intervals for subsequent gene ontology (GO) analysis.

### Putative gene mining within the clusters

The availability of the reference genome and gene annotations in cowpea enables us to identify potential candidate genes within key genomic regions. In this study, all the model genes were retrieved from Phytozome and the cowpea database (http://phytozome.jgi.doe.gov/). Using gene annotation followed by gene expression pathway and available literature, we obtained 13 candidate genes associated with seed size regulation on Vu07 and Vu011 in the cowpea genome (**Table 4**). Seven candidate genes were located on Vu07, including *Vigun07g145400*, encoding a DEAD/DEAH box helicase (IPR011545), which is implicated in ribosomal large subunit assembly and nucleic acid binding, and *Vigun07g143000*, an F-box domain-containing protein (IPR001810) involved in protein binding and targeted degradation. Also, present are *Vigun07g106800*, a serine/threonine-protein kinase (IPR002290); *Vigun07g113800*, an auxin-binding protein (IPR000526); and *Vigun07g114700*, which encodes a WD40-repeat-containing protein (IPR015943) that regulates transcription and RNA processing. Two additional genes—*Vigun07g114900*, with an ankyrin repeat domain (IPR020683), and *Vigun07g127100*, encoding a WRKY transcription factor (IPR003657)—additionally support the functional diversity of seed size regulation on this chromosome. On Vu08, two genes were retained, including *Vigun08g217500*, encoding another WRKY domain-containing transcription factor (IPR003657), and *Vigun08g217800*, a serine/threonine-protein kinase (IPR002290). These genes are likely involved in DNA-binding transcription factor activity, phosphorylation, and protein kinase activity. On Vu011, four genes were identified, such as *Vigun11g1765000* coding for F-box domain-containing protein (IPR001810), like the gene mapped on Vu07, suggesting a conserved role in protein degradation pathways. *Vigun11g178000* encodes a basic-leucine zipper (bZIP) transcription factor (IPR004827), a protein family participating in sequence-specific DNA binding and transcriptional regulation. *Vigun11g222900* possesses an AP2/ERF domain (IPR001471), another class of transcription factors critical for seed development. Finally, *Vigun11g223400* contains an RNA recognition motif (RRM) domain (IPR000504), functioning in post-transcriptional regulation via mRNA splicing.

**Table 4:** Putative genes identified that regulate cowpea seed size traits.

Chr	R-marker	L-marker	GenePos	Gene ID	Interpro ID	Putative gene	Gene functional annotation	Ref
Vu07	2_19423	2_18315	25611377-25619443	*Vigun07g145400*	IPR011545	DEAD/DEAH box helicase domain	ribosomal large subunit assembly, nucleic acid binding	(Li and Li, 2016)
			25374301-25375905	*Vigun07g143000*	IPR001810	F-box domain-containing protein	protein binding	(Zhang et al., 2019)
	2_32831	2_29015	19574853-19579341	*Vigun07g106800*	IPR002290	Serine/threonine-protein kinase	protein phosphorylation, protein kinase activity	(Ramachandiran et al., 2018)
	2_20060	2_55172	21054974-21059835	*Vigun07g113800*	IPR000526	Auxin-binding protein	regulation of DNA-templated transcription	(Schruff et al., 2006)
			21237468-21243706	*Vigun07g114700*	IPR015943	WD40-repeat-containing domain	Transcription regulation, RNA processing	(Hu et al., 2018)
			21268375-21269782	*Vigun07g114900*	IPR020683	Ankyrin repeat-containing domain	hydrolase activity	(Zhang et al., 2016)
	2_34565	2_03283	23430016-23433976	*Vigun07g127100*	IPR003657	WRKY domain	DNA-binding transcription factor activity	(Wang et al., 2021)
Vu08	2_34466	2_20561	37753103-37757433	*Vigun08g217500*	IPR003657	WRKY domain	DNA-binding transcription factor activity	(Wang et al., 2021)
			37770867-37773229	*Vigun08g217800*	IPR002290	Serine/threonine-protein kinase	protein phosphorylation, protein kinase activity	(Li and Li, 2016)
Vu011	2_08890	2_54826	38119868-38121126	*Vigun11g176500*	IPR001810	F-box domain containing protein	protein binding	(Zhang et al., 2019)
			38224211-38225036	Vigun11g178000	IPR004827	Basic-leucine zipper domain	Sequence-specific DNA binding transcription factor activity, Transcription regulation	(Mendes et al., 2013)
	2_25124	2_00076	41416205-41416721	Vigun11g222900	IPR001471	AP2/ERF domain	DNA-binding transcription factor activity, regulation of DNA-templated transcription	(Licausi et al., 2013)
			41438281-41443580	Vigun11g223400	IPR000504	RNA recognition motif domain	regulation of mRNA splicing via spliceosome, nucleic acid binding	(Lou et al., 2020)

### Allelic substitution effect and SNP markers prediction of seed size traits

Marker prediction effects of various alleles associated with seed size-related traits are presented in **Table 5** and **Supplementary Figure 2**. To enhance the analysis, chromosome short names were incorporated into the marker names to facilitate integration with the analysis software. The SNP markers that consistently identified QTLs exhibited significant allele segregation among the haplotypes. For the flanking markers Chr7-2_40009, Chr7-2_18315, Ch11-2_08890, and Ch11-2_29211, allele G emerged as the favorable allele associated with increased seed size traits in homozygous GG genotypes across all these SNP markers. In contrast, alleles A and C were identified as non-favorable, correlating with reduced seed size. Additionally, heterozygous GA and GC haplotypes also showed notable positive effects on seed size-related traits. For the flanking markers Ch7-2_51871 and Ch7-2_19423, allele T was found to be the favorable allele for increasing seed size traits in homozygous conditions, while allele C was non-favorable. For SNPs Ch7-2_20060 and Ch7-2_55172, allele C was favorable for increased seed size traits when homozygous. Finally, for the SNPs Ch7-2_29015 and Ch11-2_54826, allele A was found to be positively associated with increased seed size traits.

**Table 5:** Marker effects of flanking SNPs associated with seed size traits.

Traits	Markers	Allele 1	Allele 2	Sequence	Frequency	Adjusted probability	Adjusted significance	Overall significance
SHA	Ch7-2_40009	CC	GC	CCGC	0.48	0.22	NS	5.60E-05
		GC	CC	GCCC	0.07	0.22	NS	
		GG	CC	GGCC	0.45	8.20E-06	***	
	Ch7-2_51871	CC	TC	CCTC	0.48	0.37	NS	5.50E-04
		TC	TT	TCTT	0.00	0.38	NS	
		TT	CC	TTCC	0.45	2.50E-05	***	
HSW, ST, SW, SHA	Ch7-2_18315	AA	GA	AAGA	0.51	0.33	NS	8.50E-05
		GA	GG	GAGG	0.03	0.68	NS	
		GG	AA	GGAA	0.46	1.50E-05	***	
	Ch7-2_19423	CC	TC	CCTC	0.50	0.74	NS	1.50E-03
		TC	TT	TCTT	0.04	0.35	NS	
		TT	CC	TTCC	0.45	1.20E-04	***	
SW	Ch7-2_29015	AA	AC	AAAC	0.45	0.68	NS	8.90E-08
		AC	CC	ACCC	0.07	1.10E-03	**	
		CC	AA	CCAA	0.48	9.90E-08	***	
SHW	Ch7-2_20060	CC	CT	CCCT	0.45	8.40E-01	NS	0.0024
		CT	TT	CTTT	0.06	2.20E-01	NS	
		TT	CC	TTCC	0.50	0.00056	***	
	Ch7-2_55172	CC	CT	CCCT	0.45	0.41	NS	0.00015
		CT	TT	CTTT	0.07	0.32	NS	
		TT	CC	TTCC	0.49	2.30E-05	***	
SHL, SHW, SHA	Ch11-2_08890	AA	GA	AAGA	0.43	8.80E-01	NS	4.40E-07
		GA	GG	GAGG	0.04	2.80E-02	*	
		GG	AA	GGAA	0.53	2.40E-08	***	
	Ch11-2_54826	AA	TA	AATA	0.53	0.022	*	3.40E-07
		TA	TT	TATT	0.03	0.88	NS	
		TT	AA	TTAA	0.43	2.90E-08	***	
SHL	Ch11-2_29211	AA	GA	AAGA	0.46	0.049	*	8.60E-07
		GA	GG	GAGG	0.04	0.96	NS	
		GG	AA	GGAA	0.50	1.90E-07	***	

*, **and *** refers to statistical significance at the probability levels of 0.05, 0.01 and 0.001 respectively.

**Figure 5:**
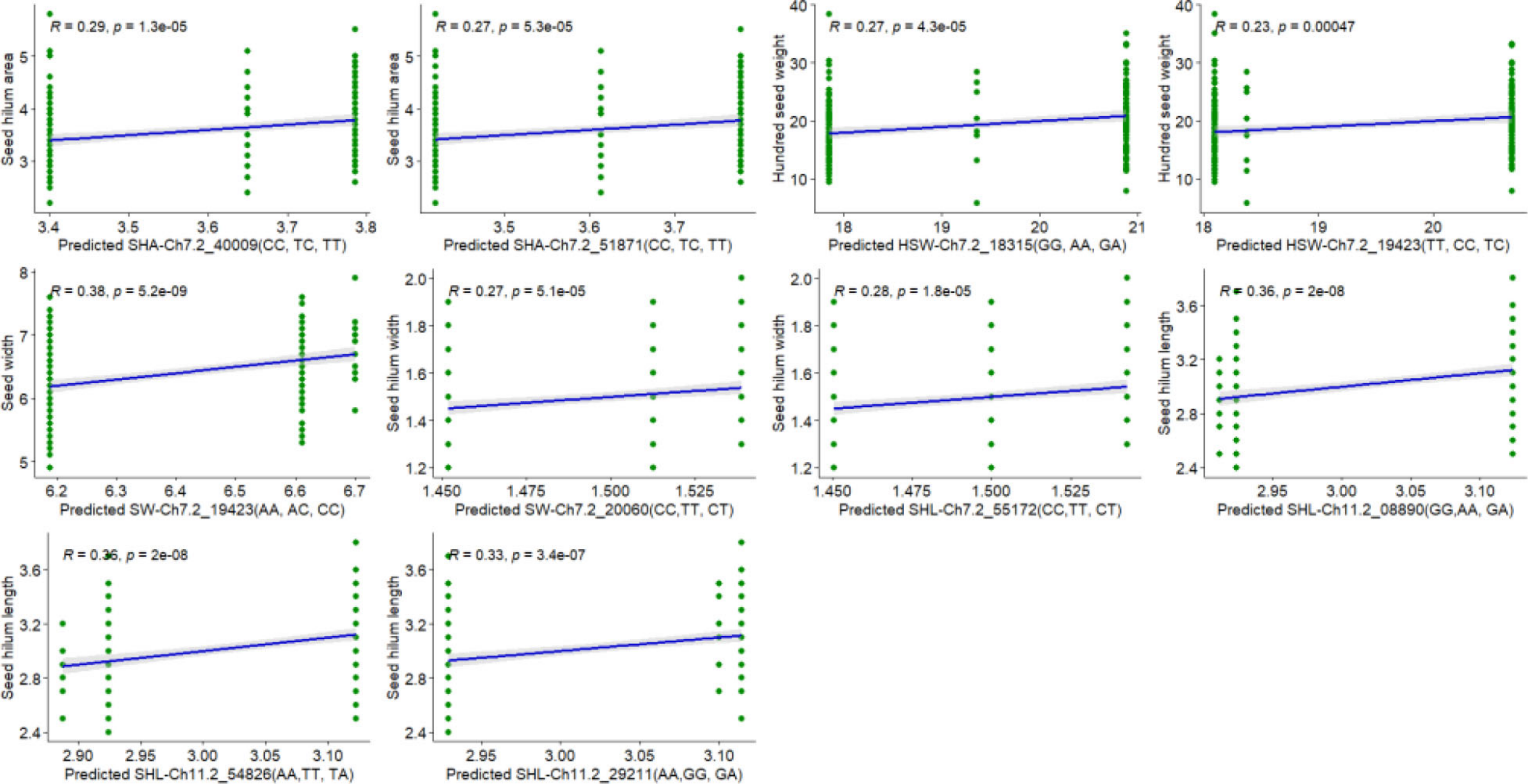
Prediction of seed size traits based on SNP markers.

Some significant markers were used to predict the performance of the 248 evaluated RILs for seed size traits (HSW, SL, SW, ST, SHL, SHW, and SHA). The predictive effects of selected SNP markers on seed size-related traits are presented in **Figure 5**. Each plot in **Figure 5** displays the relationship between SNP markers and seed size trait values, with green dots representing genotypes and the blue line indicating the fitted regression line with 95% confidence intervals. These markers exhibited significant correlations with coefficients (R) ranging from 0.23 to 0.38, indicating low to moderate prediction ability. Notably, SNP markers such as Ch7-2_40009, Ch7-2_51871, and Ch7-2_19423 showed consistent predictive trends for SHA, HSW, and SW, respectively, while Ch11-2_08890, Ch11-2_29211, and Ch11-2_54826 were strongly associated with SHL (**Figure 5**). These results suggest that the identified markers could serve as reliable genomic predictors for improving seed size traits in cowpea breeding programs.

## Discussion

Developing new cowpea varieties with higher grain yield is a main goal of cowpea breeding programs worldwide (Boukar et al., 2016; Garcia-Oliveira et al., 2020). Seed size is an important trait that contributes to grain yield and commercial value of dry grains of cowpea. At present, there are a number of cowpea varieties with a wide range of variations in seed sizes and shapes, which influence producer and consumer preferences and market value (Lucas et al., 2013). Therefore, elucidating the genetic architecture of seed seed‐size and yield related traits is important for accelerating breeding progress effectively. One powerful approach to accelerate the selection of new cowpea varieties characterized by superior traits is QTL analysis, which has enabled the identification of genomic regions responsible for the observable variability in certain important traits (Andargie et al., 2014; Lo et al., 2018; Garcia-Oliveira et al., 2020; Sodo et al., 2025).

In the present study, we evaluated, across different environments (field trials and glasshouse), a RIL population derived from a bi-parental cross, for seed and yield traits. Frequency distributions for evaluated traits did not deviate significantly from normality, confirming their quantitative inheritance. Substantial phenotypic variation was observed for seed and yield components, and positive transgressive segregation was observed for all the traits, suggesting complementary alleles contributed by both parents, and the estimated heritability across the measured traits was high (>60%). Similar findings were reported previously (Garcia-Oliveira et al., 2020; Sodo et al., 2025).

QTL analysis was carried out individually for seed size traits and in combination with yield traits to improve the accuracy and stability of QTL mapping for all traits. A total of 36 QTLs were consistently identified across the four environments used for seed size traits, while 8 QTLs were detected for yield traits. Comparison of the QTLs identified in the present study with those reported previously revealed a high level of consistency for several seed size and yield-related traits in cowpea, while also uncovering novel genomic regions controlling mentioned traits (Andargie et al., 2014; Lo et al., 2018; Garcia-Oliveira et al., 2020; Sodo et al., 2025). Although the physical positions of some QTL peaks differed between studies, their occurrence within the same chromosomal regions indicates, difference in genetic background, environmental effects, or differences in experimental design. Among these evaluated traits, hilum size has rarely been considered in the traditional breeding process. The seed hilum size in grain crops is an important trait with implications for seed development, quality, and market value. It is the point of attachment between the seed and the pod, which plays an important role in the transport of nutrients and water during seed formation and seed emergency. Its size can affect the efficiency of resource allocation into the developing seed, which may affect seed weight and composition. From a commercial perspective, hilum size, along with color, significantly impacts the visual appearance of the seed, which is a key determinant of consumer preference and market classification particularly in food-grade types (Hardham, 1976; Thorne, 1981). Breeders have not previously regarded hilum size as a key trait in their efforts to improve cowpea yield. A few years ago, a company involved in infant food production contacted IITA to access a cowpea variety with a little to no colorless hilum (Fatokun, personal communication). To date, there are no records in the literature on QTL for seed hilum traits (SHL, SHW, and SHA) in cowpea, indicating this is the very first time. However, the detection of QTLs with effects on seed hilum traits has been previously reported in soybean (Zhao et al., 2021) and has offered limited insight into potential candidate genes for seed-related traits.

The correlated responses observed between traits can be explained at the molecular level by the presence of co-localized QTLs, which may harbor genes with pleiotropic effects (Chander et al., 2008). In the present study, QTL clusters influencing multiple traits were identified on vu07 and vu011, indicating that these traits may be genetically linked or influenced by common regulatory regions. This co-localization suggests that genes within these regions could have multiple effects on different traits. Similar findings were reported by Sodo et al. (2025), and previous studies have shown that the clustering of QTLs controlling multiple traits in cowpea is relatively unusual (Garcia-Oliveira et al., 2020; Wu et al., 2024). Both parental lines contributed favorable alleles for seed size, emphasizing that beneficial alleles can also originate from parents with lower phenotypic performance; similar results were also reported earlier (Fatokun et al., 1992). Moreover, the presence of QTL clusters could signify a linkage of genes or the pleiotropic effects of a single QTL (Cao et al., 2017; Liu et al., 2017). The comprehensive analysis of QTL clusters in this study indicates that breeding programs targeting improvements in the measured traits should prioritize QTL clustering with a focus on selecting QTLs within these regions. Furthermore, the presence of QTL clusters supports evidence that certain trait-related genes are more densely concentrated in specific genomic regions of the crop’s genome compared to others (Fang et al., 2017). The co-localization of QTLs for hundred-seed weight (HSW), seed size components, and number of seeds per pod (NSP) within the Vu07 and Vu011 region suggests either pleiotropic effects of a single locus or tight linkage among closely associated genes. The negative correlation between hundred-seed weight (HSW) and number of seeds per pod (NSP) reported (r = -0.48) suggests potential trade-offs between seed size and seed number (Garcia-Oliveira et al., 2020; Sodo et al., 2025). Compared to soybean, reported studies on cowpea seed size genetics are limited (Luo et al., 2023; Zhang et al., 2024). Identifying candidate genes within the QTL regions is crucial for improving cowpea seed size (Abou-Elwafa and Shehzad, 2018; Lo et al., 2018).

The stability of the QTL is essential for use in cowpea breeding programs. Among these QTLs, eight were consistently detected across at least two environments and were defined by the same flanking markers, indicating their stability. The identification of consistent and stable QTLs across different field experiments (FY) and glasshouse (GH) suggests that these genetic regions consistently affect seed-related traits regardless of growing environments, making them suitable for marker-assisted selection in cowpea breeding programs. This observation suggests that these regions of the cowpea genome have remained conserved and are co-inherited over generations. To identify candidate genes for seed-related traits, the stable and clustered QTL regions were used as a reference to retrieve genes from the cowpea genome database (www.phytozome.net). Thirteen genes were identified on Vu07, Vu08, and Vu011 as potential candidates that are involved directly or indirectly in seed development. These identified genes encode proteins with conserved domains previously implicated in seed regulation in other crops, supporting their potential regulatory roles in cowpea. On Vu07, *Vigun07g145400* codes for DEAD/DEAH box helicase domain protein, which has been shown to play essential roles in ribosomal large subunit assembly and nucleic acid binding, cell proliferation and development, which are crucial for seed tissue growth (Li and Li, 2016). *Vigun07g143000* encodes an *F*-box domain-containing protein involved in protein degradation through the ubiquitin-proteasome pathway and has been linked to seed size control by modulating growth-related signaling proteins (Zhang et al., 2019). Similarly, *Vigun07g106800*, coding for a serine/threonine-protein kinase, regulates seed development by modulating phosphorylation cascades essential for cell division and expansion (Ramachandiran et al., 2018). Hormonal regulation also plays a critical role in seed development. *Vigun07g113800* encodes auxin-binding protein, an essential hormone for embryogenesis and seed enlargement (Schruff et al., 2006). Transcriptional control is further highlighted by *Vigun07g114700*, encoding WD40-repeat-containing protein known for grain filling via participating in transcription regulation and RNA processing (Hu et al., 2018). *Vigun07g127100* codes for the WRKY domain and functions as a DNA-binding transcription factor, likely involved in seed development through regulation of gene expression during embryo formation (Wang et al., 2021). Additionally, *Vigun07g114900*, which encodes an ankyrin repeat-containing domain protein, is reported to participate in signaling and protein-protein interactions influencing seed morphology (Zhang et al., 2016). On Vu08, *Vigun08g217500* encodes a WRKY transcription factor, reinforcing the conserved role of WRKYs domain in seed size regulation as reported by Wang et al. (2021). This gene may work in coordination with *Vigun08g217800*, a serine/threonine-protein kinase, which further indicates that kinase signaling cascades are central to seed development cycles (Li and Li, 2016). On Vu011, additional genes support the significance of transcriptional and post-transcriptional control mechanisms. *Vigun11g176500*, another F-box domain-containing gene, contributes to seed development (Zhang et al., 2019). *Vigun11g178000* encodes a basic-leucine zipper (bZIP) transcription factor, known to bind specific DNA sequences and regulate gene expression related to seed filling and maturation (Mendes et al., 2013). *Vigun11g222900 (AP2*/ERF domain) has also been demonstrated to influence seed size by acting on developmental and hormonal pathways, mainly ethylene and ABA (Licausi et al., 2013). Finally, *Vigun11g223400* (RNA recognition motif), which may affect seed traits by regulating mRNA splicing and stability during seed development (Lou et al., 2020). In summary, the functional annotations of these candidate genes indicate that seed size-related traits’ regulation in cowpea is coordinated by a network of transcription factors, signaling proteins, and RNA-binding proteins. The repeated mapping of conserved protein families such as WRKY, F-box, and protein kinases on different chromosomes suggests a polygenic and integrative control mechanism. Fine mapping and validation in larger populations will be necessary to precisely delimit candidate gene regions using higher-density SNP platforms. The analysis of SNP marker associations with seed traits in cowpea revealed significant haplotype segregation. Alleles GG, AA, CC, and TT for different selected SNPs were found to be favorable predictors for enhanced seed size traits. These findings underscore the potential of specific genetic markers and their corresponding alleles to facilitate the selection of cowpea genotypes that exhibit desirable seed sizes. The markers’ effects on seed size-related traits were assessed to evaluate their potential utility in marker-assisted selection. The results revealed significant correlations between specific SNPs and the mentioned traits. The identification of these markers offers valuable tools for breeders, particularly in the context of genomic selection and marker-assisted breeding. Further validation of the identified markers in broader germplasm panels is an important next step for marker-assisted selection. In conclusion, this study provides insights into the genetic control of seed size and yield traits in cowpea, identifies new QTLs and candidate genes, and some markers that can be deployed in breeding programs to develop high-yielding, large-seeded cowpea varieties, which are highly needed in the boiled grain market segment. Further studies, including transcriptome analysis and fine mapping with higher marker density, will be required to validate these associations and candidate genes. Validation of genotypes with favorable alleles will provide valuable breeding materials for enhancing cowpea seed size traits through marker-assisted selection in future breeding programs.

## Data Availability

The datasets presented in this study can be found in online repositories. The names of the repository/repositories and accession number(s) can be found in the article/**Supplementary Material**.
