# Supplementary material for: Genetic loci underlying seed size and yield-related traits in cowpea
Source: Front Plant Sci. 2026 Mar 16;17:1772657. doi: 10.3389/fpls.2026.1772657 (PMC13034679; doi:10.3389/fpls.2026.1772657)
Supplement: Supplementary file 1 [file DataSheet1.docx]

**Table S1:** Distribution of a total of 916 high-quality SNPs on 11 chromosomes

| **Chr** | **Nb-SNPs** | **Dis (cM)** | **Mean (cM)** |
| --- | --- | --- | --- |
| Chr1 | 79 | 56.5 | 0.7 |
| Chr2 | 38 | 42.5 | 1.1 |
| Chr3 | 118 | 87.8 | 0.7 |
| Chr4 | 82 | 54.6 | 0.7 |
| Chr5 | 93 | 89.6 | 1.0 |
| Chr6 | 63 | 50.4 | 0.8 |
| Chr7 | 96 | 84.2 | 0.9 |
| Chr8 | 97 | 73.0 | 0.8 |
| Chr9 | 73 | 83.0 | 1.1 |
| Chr10 | 69 | 44.2 | 0.6 |
| Chr11 | 108 | 45.7 | 0.4 |
| Total | 916 | 711.7 | _ |
| **Average** | **83.27** | **64.7** | **0.80** |


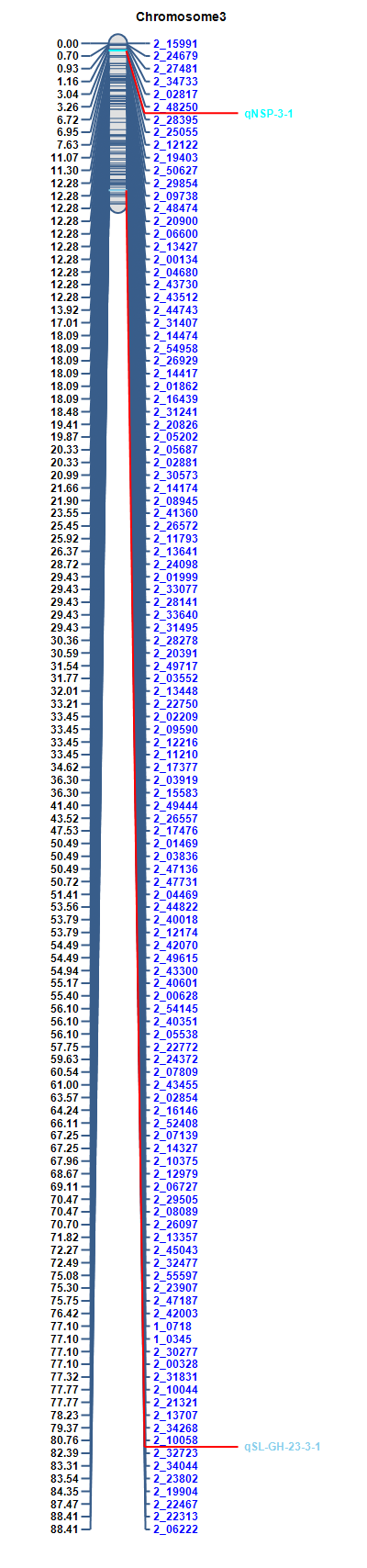

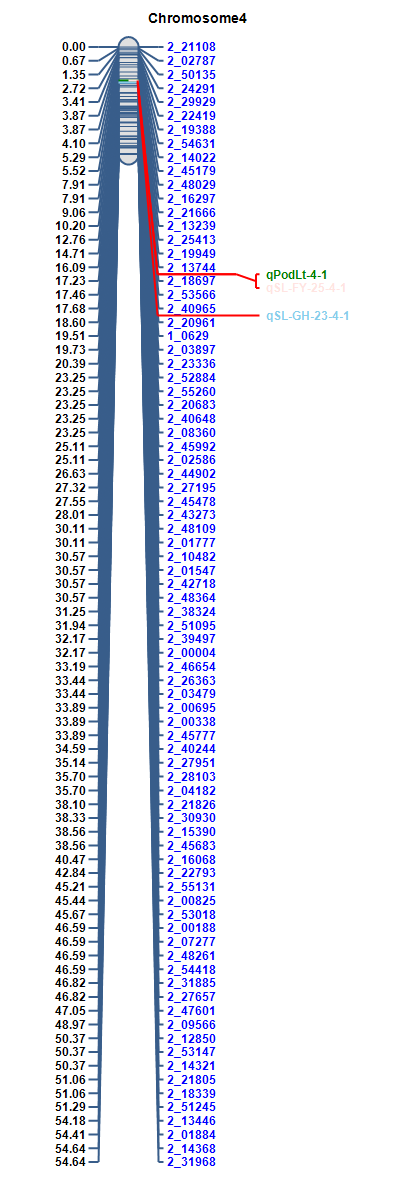

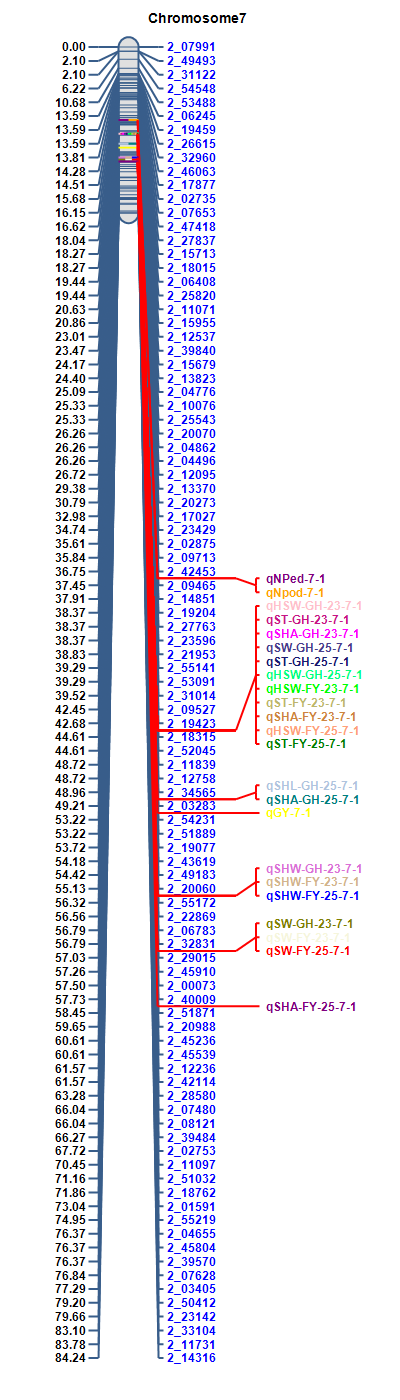

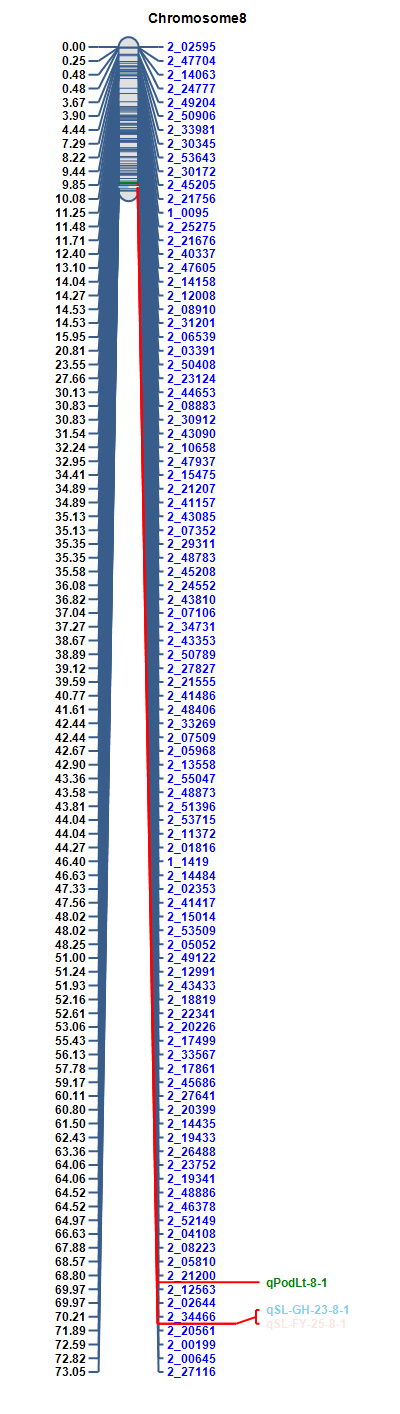

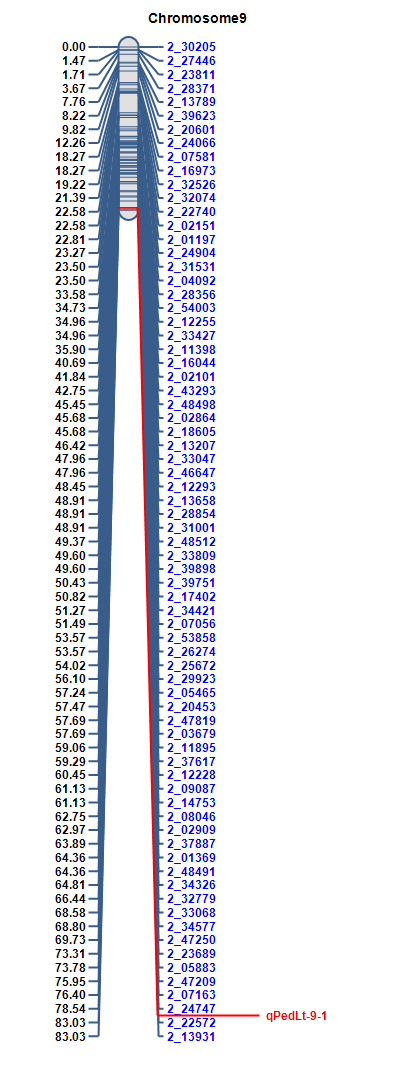

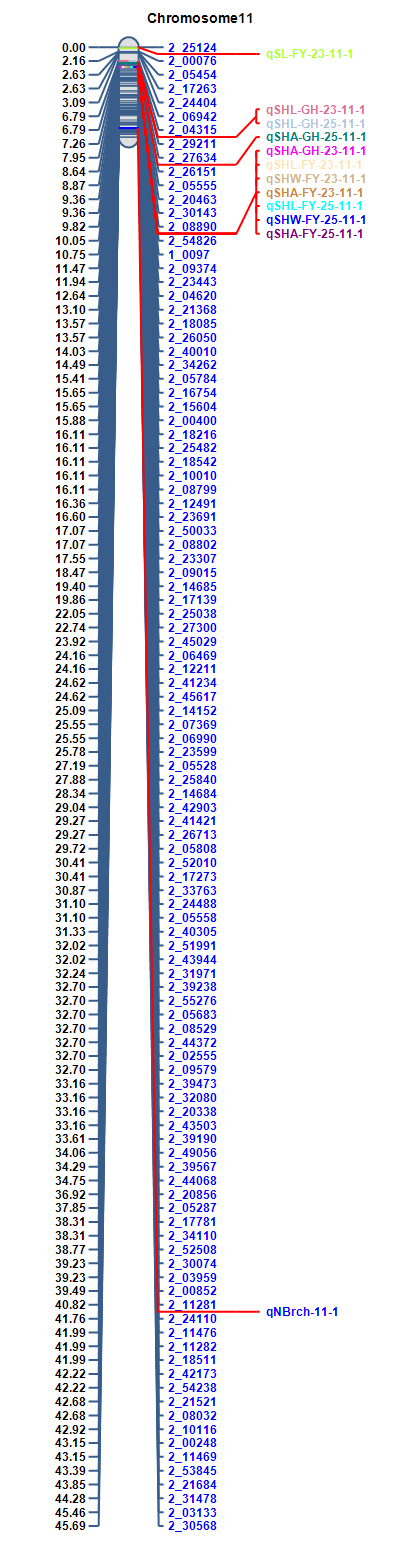


**Figure S1.** QTLs positions associated with seed size and grain yield-related traits of cowpea


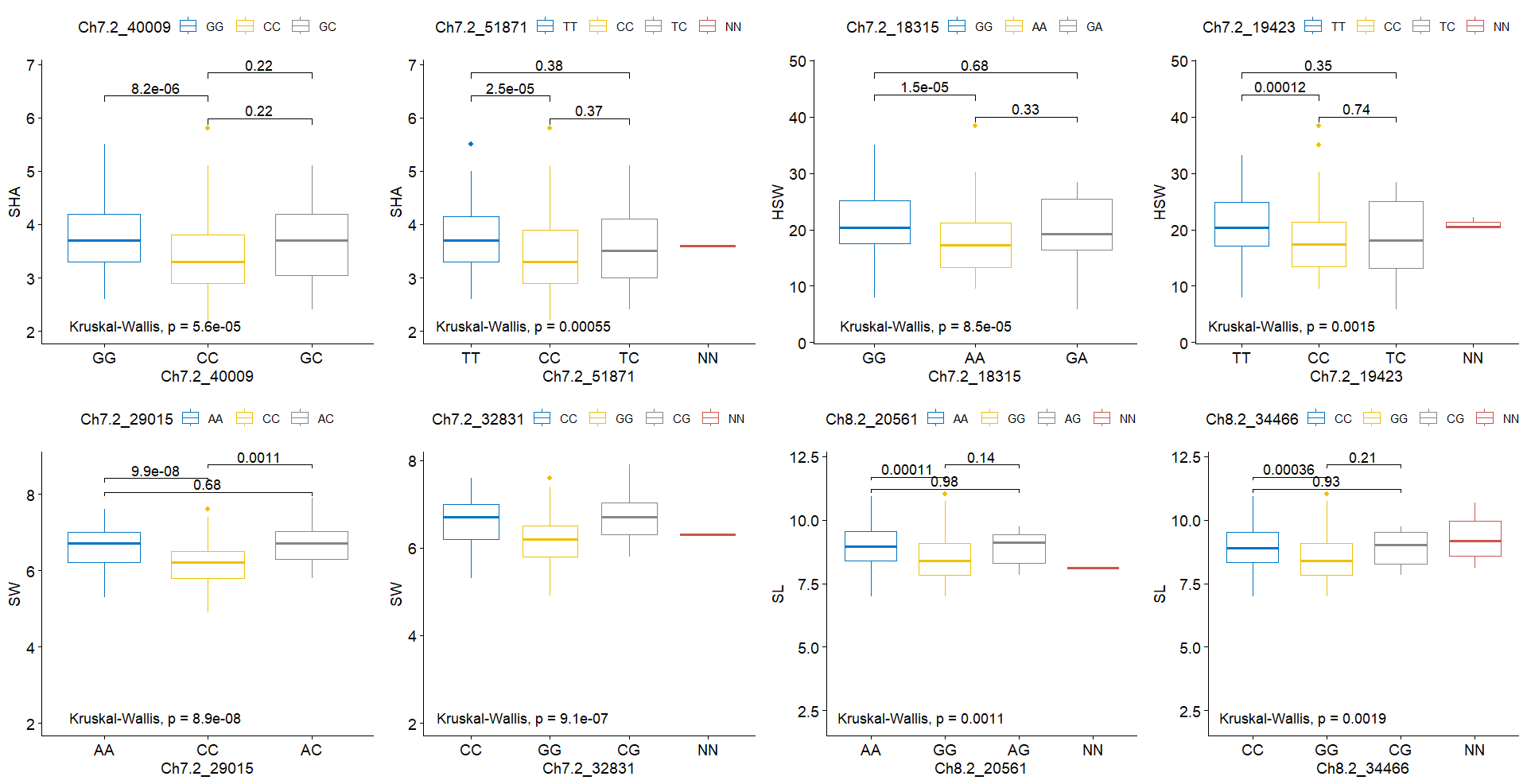

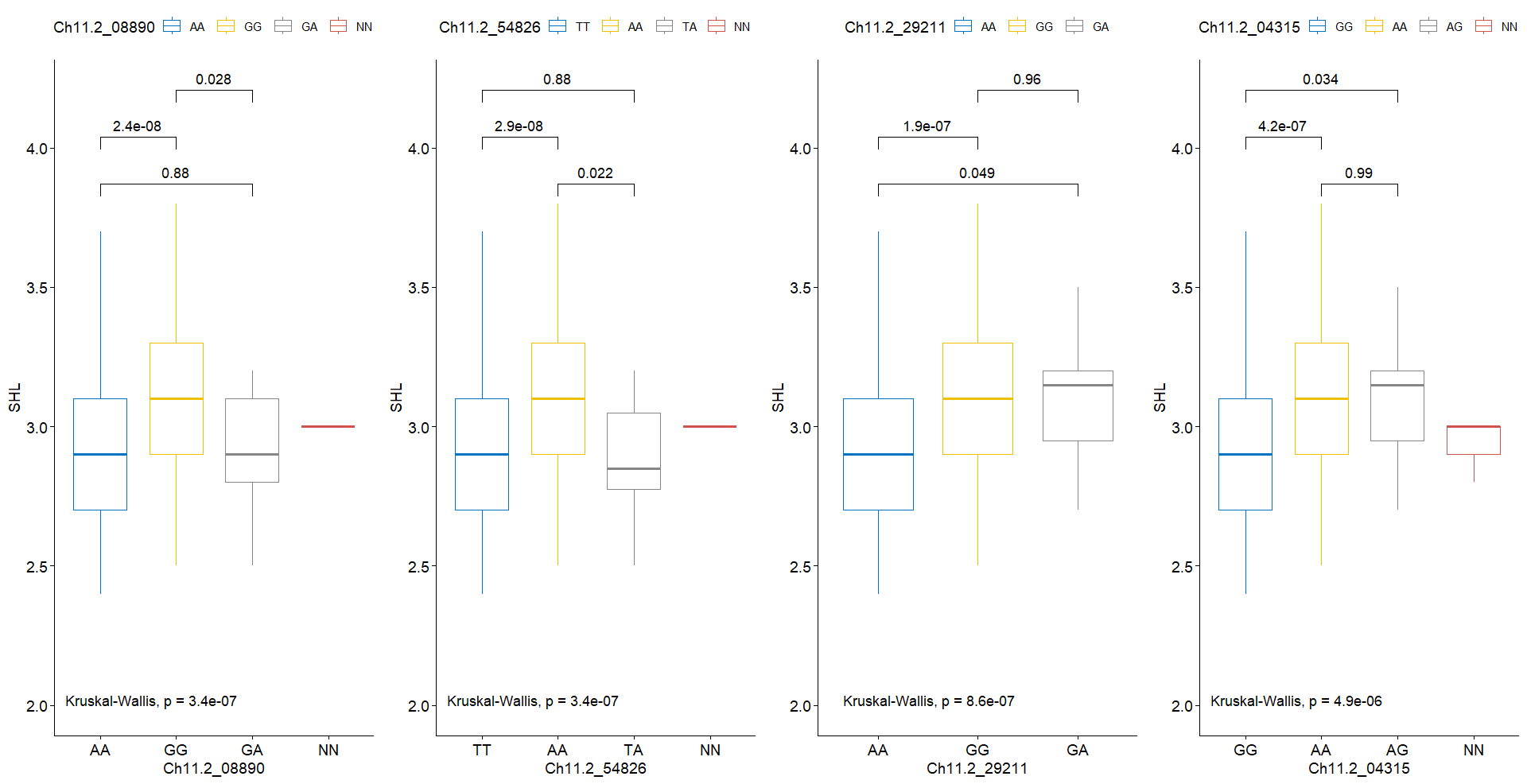


**Figure S2**. Allelic substitution effect and SNP markers prediction of seed size traits
